# Current Knowledge Attitudes, and Practices of Healthcare Providers about Leprosy in Assam, India

**DOI:** 10.4103/0974-777X.68527

**Published:** 2010

**Authors:** Sumit Kar, S Ahmad, Ranabir Pal

**Affiliations:** *Department of Community Medicine, Sikkim Manipal Institute of Medical Sciences (SMIMS) and Central Referral Hospital (CRH), 5^th^ Mile, Tadong, Gangtok, Sikkim - 737 102, India*; 1 *Department of Community Medicine, Silchar Medical College, Silchar, Assam, India*

**Keywords:** Attitudes, Behavior, Healthcare providers, Leprosy

## Abstract

**Background:**

Leprosy is a chronic infectious disease that is associated with serious morbidity and is a disease of public health concern because of the case load and the social stigma attached to the disease.

**Aim:**

To understand the knowledge of, and attitudes towards, leprosy amongst healthcare providers in Assam, India.

**Settings and Design:**

This cross-sectional study was conducted during March to June 2007 in different health institutions of the Kamrup district of Assam.

**Results:**

Among the program managers interviewed, only half were organizing training sessions, and 37.5% were involved in supervision of the program activities at the periphery level. Among the program managers who were involved with leprosy elimination, only half were organizing training session and 37.5% were involved in supervision of the program activities at the periphery level. Medical officers consistently demonstrated higher knowledge about leprosy, followed by health supervisors and multipurpose workers (MPWs), including nursing staff. Regarding training status with regard to leprosy, 90% of medical officers, 80% of health supervisors and around 87% of MPWs (83% of male MPWs and 89% of female MPWs) had attended training programs on leprosy. Regarding WHO MDT, 80% of health supervisors, 84.8% of male MPWs and 86.2% of female MPWs had an idea of MDT and treatment duration of various categories of patients.

**Conclusions:**

These observations suggest that there appear to be adequate knowledge and positive behavior among healthcare providers with regard to leprosy in this part of India. However, there is still a need to organize training programs at regular intervals to train new recruits, as well as reinforce and update the knowledge of those already trained.

## INTRODUCTION

Leprosy is still prevalent in certain parts of the world, particularly India and South America.[1] Social stigma and prejudices associated with leprosy still remain major obstacles for its eradication.

After India had declared the goal of elimination of leprosy, it was no longer cost effective to rely on rapid surveys, population surveys and contact tracing for case detection, and since then most new cases have been diagnosed by the General Health Service staff; and since 2001, MDT services were integrated with the general healthcare delivery system.[2]

Human resource development of general healthcare system (GHS) is a vital preparatory action for successful integration of leprosy into GHS. District-level technical support teams (DTSTs) have been formed with responsibility for building up the capacity of medical and paramedical staff working at various levels of the system.[3]

Studies indicated that in the currently declining phase of leprosy endemicity, employing a conventionally trained, salaried class of paramedical staff for field surveys is prohibitively expensive in view of the high cost per case detected. Involving primary healthcare workers and community-derived workers is cost effective.[4]

Integration of the vertical leprosy program with the existing horizontal health program poses various administrative and operational challenges for program managers.[5]

In this context, it is necessary to know the current levels of knowledge, attitude and practices (KAP) prevailing among healthcare personnel with regard to leprosy at a given point in time, so that required knowledge and skills can be imparted. This study was carried out to identify the health-providing behavior of healthcare personnel working in an endemic region of Kamrup district of Assam under the MDT-implementation and leprosy-elimination program.

## MATERIALS AND METHODS

This cross-sectional study was based on both quantitative and qualitative parameters with review of relevant documents, records and literature at Kamrup district of Assam, India, from March 2007 to June 2007. To assess the training status and program management at the community level, it was decided to interview the medical officers and health workers working in various health institutions in the district. The district has two divisions: urban and rural. Rural Kamrup had 14 block primary health centers (PHCs). Keeping in view the operational feasibility and manpower constraints, we took up about 25% of these PHCs, i.e., four. These four PHCs were selected randomly from the set. The following block PHCs got selected, viz., Uparhali block PHC, Boko block PHC, Sonapur block PHC and Hajo block PHC.

For urban representation, we had taken into consideration major government health institutions. The Upgraded Urban Leprosy Centre attached to the dermatology clinic of Guwahati Medical College, and the Urban Leprosy Centre attached to Mohendra Mohan Hospital (MMH) were the primary sources of data.

Secondary data, i.e., data regarding the manpower involved in delivering healthcare in these selected centers, was collected from the office of the Director of Health Services.

The data collection tool used for the study was an interview schedule that was developed at the institute with the assistance from the faculty members and other experts. Different information modalities were sought from different groups of health personnel. The questionnaire was discussed among the faculty of Department of Community Medicine, Guwahati Medical College, and it was also shared with the zonal officers of the district dealing with leprosy. The schedule was then pre-tested in the field to rule out operational constraints. The pilot study was carried out at the institute among general healthcare personnel, following which some of the questions from the interview schedule were modified.

This pre-designed and pre-tested questionnaire contained questions relating to the information on designation of the staff; his/ her training status in leprosy; the presence or absence of knowledge about leprosy, its categorization, its treatment modalities; and individual involvement in the National Leprosy Eradication Programme The study was approved by the institutional ethics committee of Guwahati Medical College. All the healthcare providers present during the study period were included. Those on leave or transferred to another district were excluded from the study. Accordingly, 50 medical officers, 40 health supervisors, 66 male multipurpose health workers and nonmedical assistants, 65 female multipurpose health workers and auxiliary nurse midwives, along with 8 policy makers at the district headquarters, were selected for study. All the participants were explained about the purpose of the study and were ensured strict confidentiality, and then informed consent was taken from each of them. They were given the option of not participating in the study if they so desired.

## Statistical analysis

The data collected were thoroughly screened and entered into MS-Excel spreadsheets, and analysis was carried out. The procedures involved were transcription, preliminary data inspection, content analysis and interpretation. Percentages were used in this study to analyze study variables.

## RESULTS

Among the program managers interviewed, only half were organizing training sessions, and 37.5% were involved in supervision of the program activities at the periphery level [Table 1].

**Table 1:** Knowledge, attitude and behavior of the district-level policy makers of National Leprosy Eradication Programme (NLEP) with regard to leprosy

Health-Providing behavior	Policy makers (*n* = 8)	%
Involvement in leprosy elimination campaign	8	100.0
Knowledge about NLEP	8	100.0
Knowledge about diagnosis and treatment	8	100.0
Knowledge about MDT stock	4	50.0
Organizing training sessions for leprosy	4	50.0
Supervising anti-leprosy activities	3	37.5

Regarding training status in leprosy, 90% of medical officers, 80% of health supervisors and around 87% of MPWs (83% of male MPWs and 89% of female MPWs) had attended training programs on leprosy [Table 2].

**Table 2:** Leprosy training status of generalhealthcare system staff (GHCS)

Category of GHCS staff	Number of personnel interviewed (*n* = 8)	Number and percent of personnel trained in leprosy	
		No.	(%)
Medical officers	50	45	90%
Health supervisors	40	32	80%
MPWs (male) and NMA*	66	55	83%
MPWs (female) and ANM**	65	58	89%

* NMA–Non-medical assistant;

** ANM-Auxiliary Nurse Midwife

The health-providing behavior of the program-implementing personnel was also studied. Sixty percent of the medical officers had a role to play in Information Education Communication (IEC). Among the health supervisors, 90% were aware of the cause of leprosy, while 92.5% were aware of the diagnosis, categorization of patients and treatment according to category. All the health supervisors knew about MDT, and 80% of them had an idea about the program. Seventy-five percent of them played a role in IEC campaign. Among male grass-root level multipurpose workers, 83.3% were aware of the cause of leprosy; 87.9% could diagnose the disease; 84.8% were aware of MDT, categorization of patients and their treatment duration; 81.8% had knowledge of the program; and 78.8% were involved in IEC campaigns. Among female MPWs, 66.2% were aware of the cause of the disease; 87.7% could diagnose the disease; 86.2% had an idea of MDT and treatment duration of various categories of patients; 80% of respondents could categorize the patients; 76.9% knew about the program; and 90.7% of the respondents had a role to play in IEC campaigns [Table 3].

**Table 3:** Knowledge, attitude and health-providing behavior of the general healthcare staff with regard to leprosy

Health-Providing behavior	Categories of program-implementing personnel							
	Medical officers (*n*=50)		Health supervisors (*n*=40)		MPWs (male) and NMA (*n*=66)		MPWs (female) (*n*=65)	
	No.	%	No.	%	No.	%	No.	%
Cause of leprosy	50	100	36	90.0	55	83.3	43	66.2
Diagnosis of leprosy	50	100	37	92.5	58	87.9	57	87.7
Knowledge of MDT	50	100	40	1000	56	84.8	56	86.2
Knowledge of NLEP	50	100	32	80.0	54	81.8	50	76.9
Patients’ categorization	50	100	37	92.5	56	84.8	52	80.0
Duration of treatment according to category	50	100	37	92.5	56	84.8	56	86.2
Role played in IEC	30	60	30	75	52	78.8	59	90.7

(Multiple responses/ choices applied)

## DISCUSSION

Our study revealed a diverse yet good knowledge and attitudes towards leprosy among the ‘MDT (multidrug therapy)’-implementing personnel. Medical officers consistently demonstrated higher knowledge about leprosy in comparison with health supervisors and multipurpose workers, including nursing staff.

In a study from Hyderabad city, conducted in Government Health Services dispensaries in Hyderabad in order to assess KAP and some operational parameters, medical officers consistently demonstrated higher knowledge about leprosy, followed by nursing staff and paramedical workers. More than half of the study subjects did not have specific training in leprosy.[3]

A study was undertaken as part of operational research by the Ministry of Health and Family Welfare, Government of India, to assess the level of integration of leprosy services into general healthcare system in 24 low or moderately endemic states/ union territories. The data in this study included training status of general healthcare staff with regard to leprosy. About half (53.2%) of the existing medical officers, 83.9% of health supervisors and 96.8% of multipurpose workers were found to be trained in leprosy. The study emphasized the need for training; as only 31.3% of medical officers were able to diagnose leprosy, and most of them were relying on vertical staff and skin specialists for confirmation.[6]

Assam reported a higher level (97%) of training of medical officers in leprosy. Training of health supervisors and multipurpose workers was better than that of medical officers in most of the states. Tripura reported negligible training of all the health functionaries because of specific local problems. In Assam and Maharashtra, medical officers in all (100%) health facilities were diagnosing and treating leprosy cases, as compared with Himachal Pradesh (30%). However, lower involvement of GHS staff in recording and reporting was noted in Assam (both 0%, respectively), Andhra Pradesh (10% and 30%, respectively).[7]

An investigation into the attitudes, beliefs and behavior of 730 primary healthcare (PHC) workers with regard to MDT was carried out in Yangzhou and Dongtai districts of China, which revealed that only half of the PHC workers had a basic knowledge of MDT and a desire to participate in MDT implementation.[8]

The knowledge and attitude of health workers in northwestern Botswana with regard to leprosy were determined by interviewing 99 health workers from various health institutions. Knowledge on causation of leprosy was generally lacking. Although majority of respondents knew that the disease is curable, less than half knew the correct duration of treatment.[9] A study conducted by the Regional Leprosy Training and Research Institute, Lalpur, Raipur, Chhattisgarh, India, revealed that 45% of medical officers, 71% of health supervisors and 75% of multipurpose workers were trained in leprosy.[10]

## CONCLUSION

To sum up, these observations suggest that there appear to be adequate knowledge and positive behavior of healthcare providers with regard to leprosy in this part of India. However, there is still a need to organize training programs at regular intervals to train new recruits, as well as reinforce and update the knowledge of those already trained. In view of the changing logistics, it is very necessary that manpower training and reinforcements should be given serious consideration by health planners.

## References

[1] Briden A, Maguire E (2003). An assessment of knowledge and attitudes towards amongst leprosy/ Hansen’s disease workers in Guyana. Lepr Rev.

[2] Chen S, Han C, Li B, Zheng R, Zhang L (2000). A survey on knowledge and skills in the early diagnosis of leprosy in general health services at different levels in Shandong Province, The People’s Republic of China. Lepr Rev.

[3] Rao PV, Rao SL, Vijayakrishnan B, Chaudhary AB, Peril S, Reddy BP (2007). Knowledge, attitude and practices about leprosy among medical officers of Hyderabad urban district of Andhra Pradesh. Indian J Lepr.

[4] Naik SS, Ganapati R (1998). Socioeconomics of a global leprosy eradication programme. Pharmacoeconomics.

[5] Raju MS, Dongre VV (2003). Integration of the leprosy programme into primary health care: A case study of perceptions of primary health care workers. Indian J Lepr.

[6] Pandey A, Patel R (2005). Integration of leprosy control into general health care system: Observations from a state with low endemicity. Indian J Lepr.

[7] Pandey A, Patel R, Rathod H (2006). Inter-state variations in integration of leprosy services into general health system in low/ moderately endemic states of India. Indian J Lepr.

[8] Chen XS, Ye GY, Jiang C, Li WZ, Bian J, Wang H (1997). An investigation of attitudes, beliefs and behaviour of leprosy patients, family members and PHC workers towards multidrug therapy in Yangzhou and Dongtai Districts of China. Lepr Rev.

[9] Kumaresan JA, Maganu ET (1994). Knowledge and attitude of health workers towards leprosy in north-western Botswana. East Afr Med J.

[10] Pandey A, Patel R, Uddin MJ (2006). Leprosy control activities in India: Integration into general health system. Lepr Rev.

